# The accuracy of digital impression with different intraoral scanners on maxillary all on four implants: an in vitro study

**DOI:** 10.1186/s13104-025-07235-x

**Published:** 2025-04-22

**Authors:** Hesham M. El-Refay, Medhat Sameh Abdelaziz, Nora M. Cheta, Mohamed F. Abdallah

**Affiliations:** 1 Department of Oral Implantology, Faculty of Dentistry, Cairo University, Cairo, Egypt; 2 Faculty of Oral and Dental Medicine, Prosthodontics Department, Future University in Egypt, Cairo, Egypt; 3 Faculty of Dentistry, Prosthodontics Department, Cairo University, Cairo, Egypt; 4 Faculty of Dentistry, Prosthodontics Department, El Galala University, Suez, Egypt

**Keywords:** Intra-oral scanning, Implants, All on four, Digital impression, Full arch implants

## Abstract

**Objectives:**

This study aimed to evaluate the effect of 30° angulation of (All-on-four) implants on the accuracy of digital impressions using different intra-oral scanners in the maxillary edentulous arch in terms of trueness and precision.

**Materials and methods:**

A maxillary completely edentulous model was 3D printed with four-cylinder holes measuring 4.3*10 mm, creating space for implant analogs at the canines and second premolar areas. The two anterior implants were placed parallel to each other with 0-degree angulation at the site of the canines, while the two posterior implants were placed at the site of the second premolars with 30° distal angulations. Four peek scan bodies were screwed to analogs. The model is scanned using an EOS X5 desktop scanner and set as a reference model. Afterward, three groups of intraoral scanners group 1 (Trios3shape), group 2 (Medit I700), and group 3 (Launca DL-202) were used to scan the model. Seven scans of the model were performed for each scanner following the manufacturer protocol. The trueness and precision of each intraoral scanner were virtually tested using the Gemoagic Control X software program.

**Results:**

Regarding trueness, there was a statistically significant deviation between the three intraoral scanners recording 38, 44, and 229 μm for the Trios, Medit I-700, and Launca scanners, respectively, while there was no statistically significant difference in precision between the Trios and Medit I700 scanners.

**Conclusions:**

The Trios scanner was the most accurate regarding trueness and precision for recording the maxillary full arch implants, followed by the Medit I-700, and the Launca scanner due to the ability of the Trios scanner to scan the posterior angulated implants as accurately as the anterior straight ones. Also, the scanner technology separately doesn’t affect scanning accuracy, but other factors should be taken into consideration such as scanner design and scanner head size.

**Clinical relevance:**

The type of intraoral scanner used in full arch cases greatly affects the accuracy of digital impressions, which may affect the fit of future prostheses, so the operator should carefully choose the proper optical scanner.

## Introduction

Despite the revolution of restorative dentistry, the rate of edentulous patients has not reduced due to the increased age of societies [1–3]. Implant placement in the maxillary arch has many limitations due to horizontal and vertical bone resorption, specifically in the posterior area due to sinus pneumatization [4, 5]. The all-on-4 concept was introduced by Paulo Malo in 1998 to restore a fully edentulous arch with only four implants and to provide immediate loading temporary prosthesis [6]. 

The All-on-4 concept includes the placement of two implants in the anterior area of the arch in the axial position and two posterior implants (one implant on each side) placed anterior to the vital structure (the mental nerve in the mandible-maxillary sinus in the maxilla) and tilted distally to enhance support for prosthesis [7, 8]. the anterior implants are usually placed at the position of the canines. In contrast, the two posterior implants are usually placed at the second premolar area at either a 30 or 45-degree angle [9]. The use of tilted implants decreases cantilever forces in the posterior area [10], and allows the use of a prosthesis consisting of 12 units despite the anterior implant position [11]. 

The conventional dental impression is defined by the Glossary of Prosthodontics as a negative replica of the surface of an object [12]. The aim of the impression step in dental implantology is to transfer the relationship between the implant or implant abutment with other oral structures. This is done using impression coping, which is attached to the implant or implant abutment [13, 14]. 

The digital impression converts intra-oral structures into virtual replicas. The accuracy of this step is very important and may determine the success of the whole treatment, as it’s very important to transfer implant position, angulation, and depth correctly to the computer-aided design (CAD) software program. Any further misfit may lead to mechanical and biological complications with restoration failure at the end [15]. Digital implant impressions are superior to traditional impressions in that there is less chance of distortion throughout the clinical and laboratory stages by eliminating conventional tray selection, dimensional changes of conventional impression, cast pouring, and its related distortion. Digital impression also eliminates laboratory transportation and the need for dental cast storage [14]. Also, there is better patient comfort and acceptability for digital impressions as it is very suitable for patients with higher gag reflexes and those allergic to different impression materials [14, 16, 17]. 

With the widespread and continuous development of intra-oral scanners (IOS), the market has wide varieties of IOS with different characteristics (acquisition methods and reconstruction algorithms) [18, 19]. 

Intra-oral scanners depend on different acquisition technologies for optical scanning, for example the Trios 3 (3-Shape, Copenhagen, Denmark) depends on confocal microscopy technology, the Medit I700 (MEDITcorp.23, Seoul, Korea); and the Launca (DL-202 Guangdong Launca Medical Device Technology Co., Ltd. Resources, China.) depends on triangulation technology [18]. 

The optical impression for all on four edentulous arches is quite difficult, especially for tilted implants, due to the absence of reference areas(teeth) in the completely edentulous jaws which affects the stitching procedure negatively also the presence of mobile tissue and saliva along with the pink color of the soft tissue are quite challenging factors in the full arch scanning. The dimension, colors, and design of the scan body along with the implant position and accessibility also affect the full arch implant scanning [20, 21]. Also, the use of different intra-oral scanners seems to have a significant effect on the accuracy of recording implant position [19]. 

Intra-oral scanner accuracy is determined mainly through trueness and precision. Trueness is the degree to which the test findings closely resemble the widely accepted reference value. While precision refers to the reproducibility of intraoral digital scans taken under the same scanning settings [22]. 

Therefore, the aim of this study was to compare the accuracy in terms of trueness and precision of three different intra-oral scanners with different acquisition technologies in the edentulous maxilla with the (All-on-four) concept. The null hypothesis was that there would be no significant difference between the three intra-oral scanners in the (all on four) digital implant impression.

## Materials and methods

### Working cast construction

A ready-made maxillary edentulous arch model (Dentsply Sirona Inc.) was scanned using an Eos x5 extra-oral laboratory scanner (Dentsply Sirona, Charlotte, NC, USA) to obtain a standard tessellation language file (STL file).

**Fig. 1:**
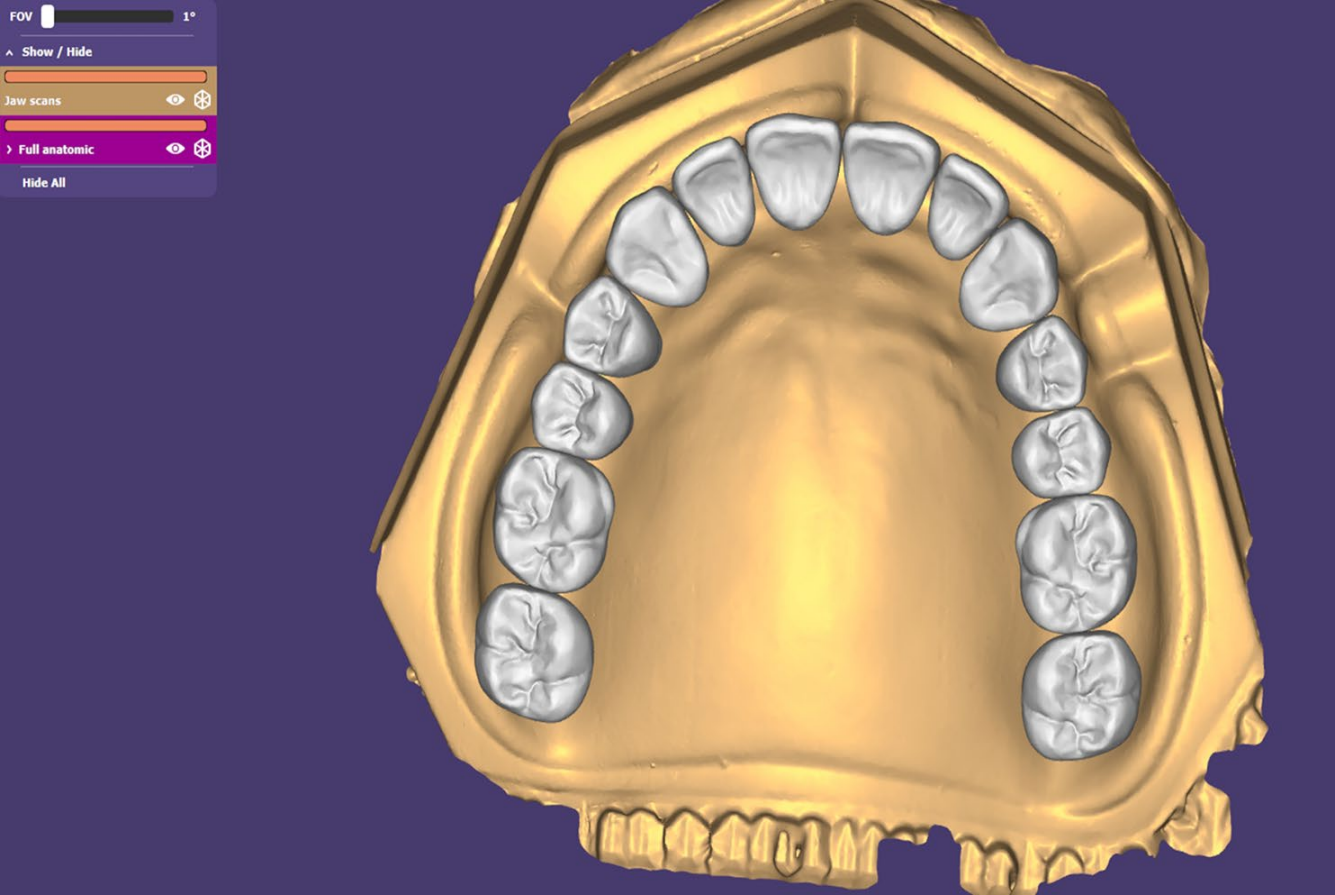
Virtual setting of artificial teeth on the maxillary edentulous model to determine the implant position according to the prosthetically driven implant placement concept

The STL file of the model was imported to a dental computer-aided design software program (Exocad GMBH, Darmstadt, Hessen, Germany) for virtual teeth setting [2, 23]., which will help to determine the implant position according to the prosthetically driven implant placement concept at the canine and premolar areas. (Fig. 1)

**Fig. 2:**
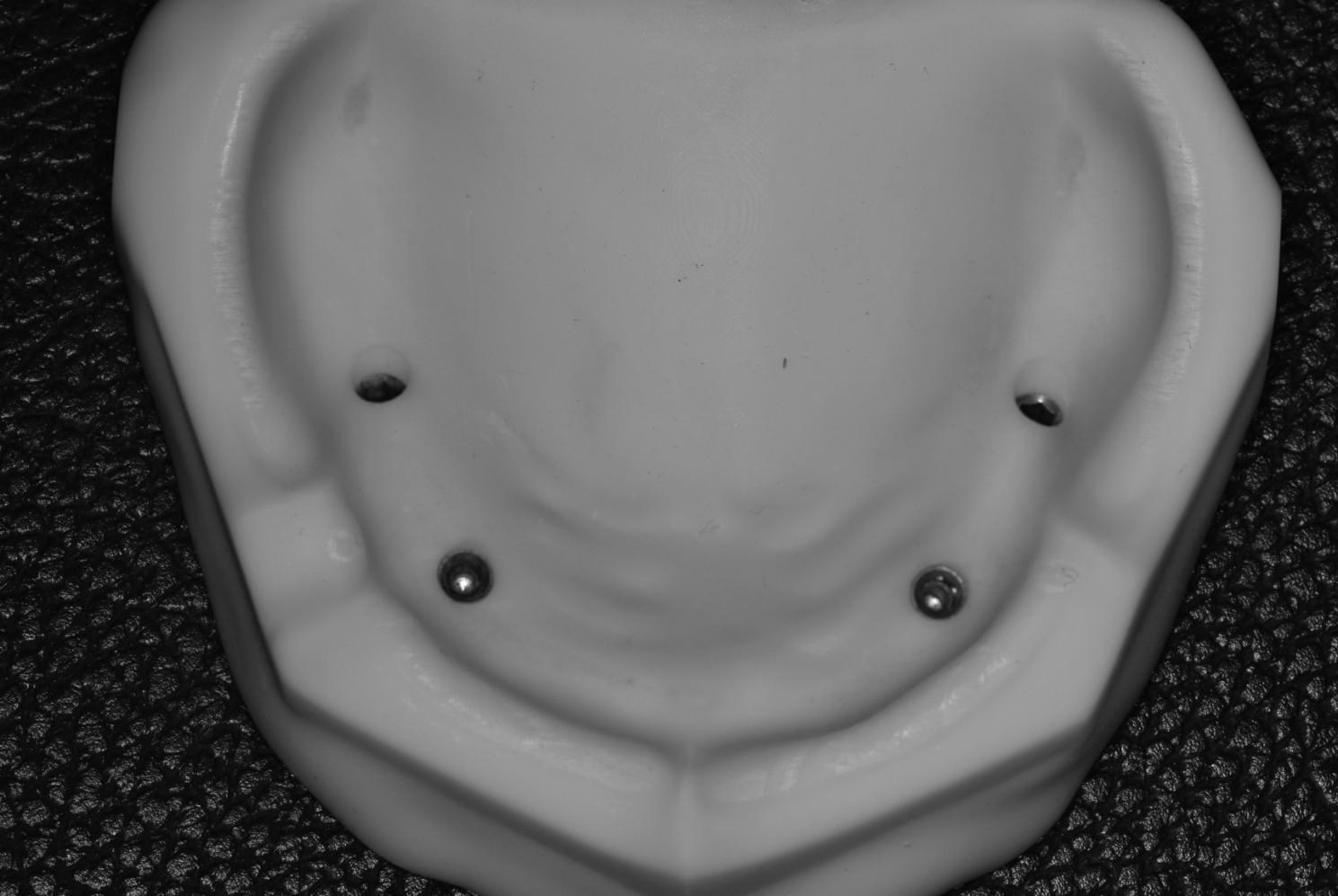
The 3D printed model with four cylindrical holes with the implant analogs attached to it

Four cylindrical holes to accommodate four implant analogs with 4.3 mm diameter and 10 mm depth were prepared virtually in the model using a free-form designing software program (Meshmixer, Autodesk Inc., San Rafael, CA, USA). The designed model was 3D printed in dental model resin by Elegoo Mars 2 (©ELEGOO, INC. 2023, Silicon Valley, Shenzhen, China). (Fig. 2)

Finally, four implant analogs (Implant Direct™ Dentistry, Legacy Implant, California, USA) (4.3 mm width x 4.3 mm platform) are cemented to the 3D printed model using resin cement (G-cem Hongo, Bunkyo-ku, Tokyo, JAPAN). The analogs were placed approximately 2–3 mm below the crest to mimic soft tissue thickness. The two anterior implant analogs were inserted at the right and left canines with zero angulation and parallel to each other, and the two posterior implants were inserted at the 2nd premolar area with 30° distal angulations.

**Fig. 3:**
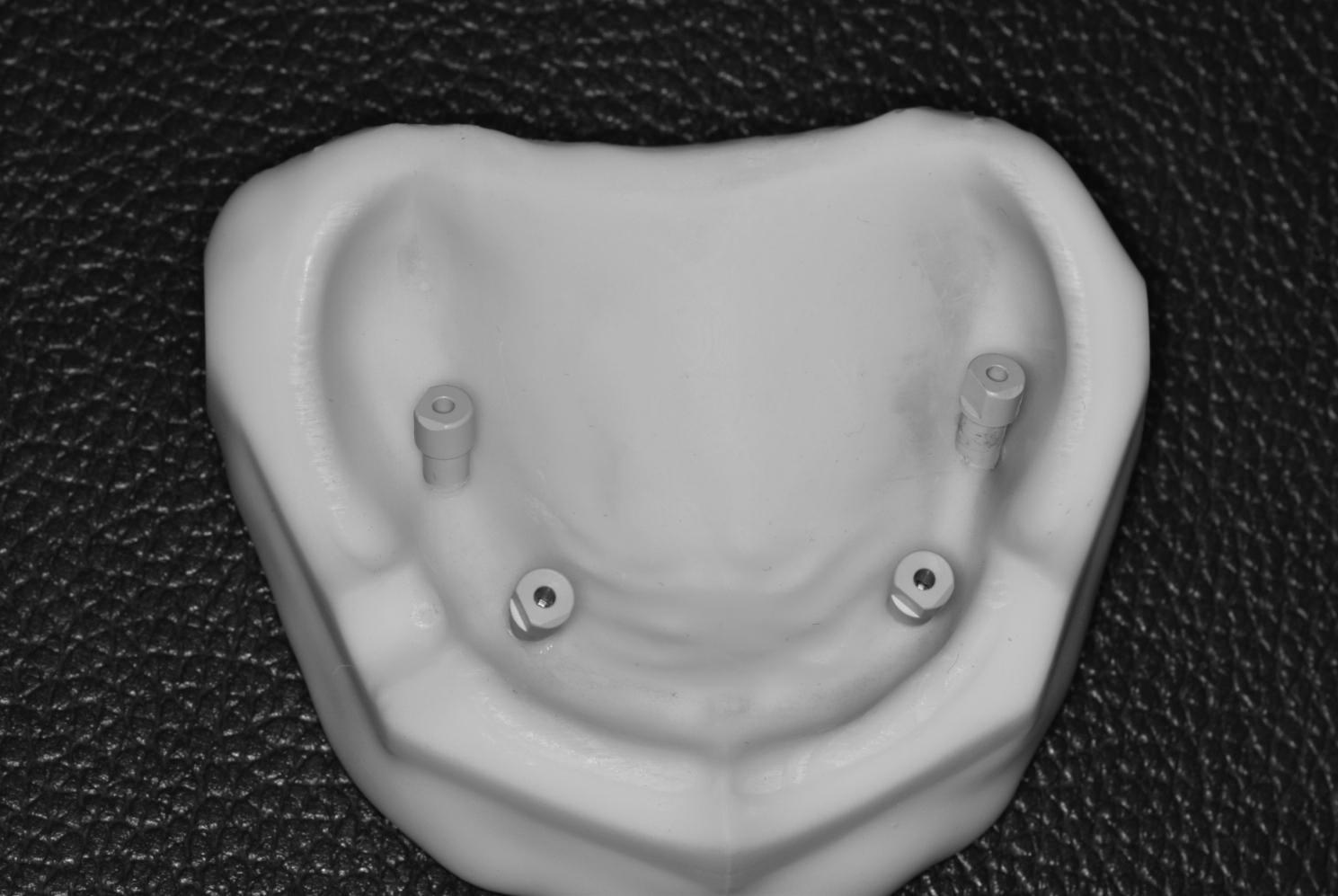
The 3D printed model with four scan bodies attached to the implant analogs

Four PEEK scan bodies (Implant Direct™ Dentistry, Legacy Implant, California, USA) were connected to the implant analogs by a screwdriver according to manufacturer instructions [24]. (Fig. 3)

### Scanning protocols

#### Comparator: extraoral scanner

The model with the scan bodies attached to it was scanned using an Eos X5 extra oral optical scanner (Dentsply Sirona, Charlotte, NC, USA), and the data was exported in STL format and set as a reference for all the upcoming comparisons [25]. 

#### Intervention: three intra-oral scanners IOSs

Three IOSs, 1-Launca (DL-202 Guangdong Launca Medical Device Technology Co., Ltd. Resources, China.), 2- Trios 3Shape (Trios 3, 3-Shape, Copenhagen, Denmark), and 3- Medit I700^®^ (MEDITcorp.23, Seoul, Korea) were used to scan the model. The model was scanned seven times, with each scanner following the same scanning protocol, starting from the occluso-palatal surfaces of the maxillary right tuberosity, moving toward the opposite side of the arch, always including two surfaces (occlusal and palatal), and finalizing the scan at the buccal side [14, 18]. 

The scanner was held between 5 and 30 mm away from the surface being scanned. Each scan time was within a small range of seconds 90–150 s. The maxillary tuberosity and rugae area were considered reference areas during the scanning procedure [26]. all the scans were performed by one experienced operator under a standard room light condition with 1003 lx [27]. 

Each scan of Trios 3-shape took about 90 s to 120 s, the Medit I-700 was done within 90 s to 140 s, and the Launca scan was performed in about 100 s to 150 s.

### Trueness and precision measurement

The accuracy of the optical scans was performed using reverse engineering software (Geomagic Control X, GOM GmbH, Germany) [14, 25]. The STL file of the desktop scanner was set as a reference, which was later segmented into two parts. The first segment consists of the four scan bodies (comparative aspect), and the other segment, composed of the edentulous arch, tuberosity, and rugae area, was used for the superimposition procedure.

For measuring the trueness of each IOS, the STL files obtained from each intra-oral scanner were superimposed onto the reference scan obtained from the desktop scanner. While measuring the precision of each IOS, the STL files obtained from each IOS were compared in pairs by setting the first scan as a reference [14]. 

**Fig. 4:**
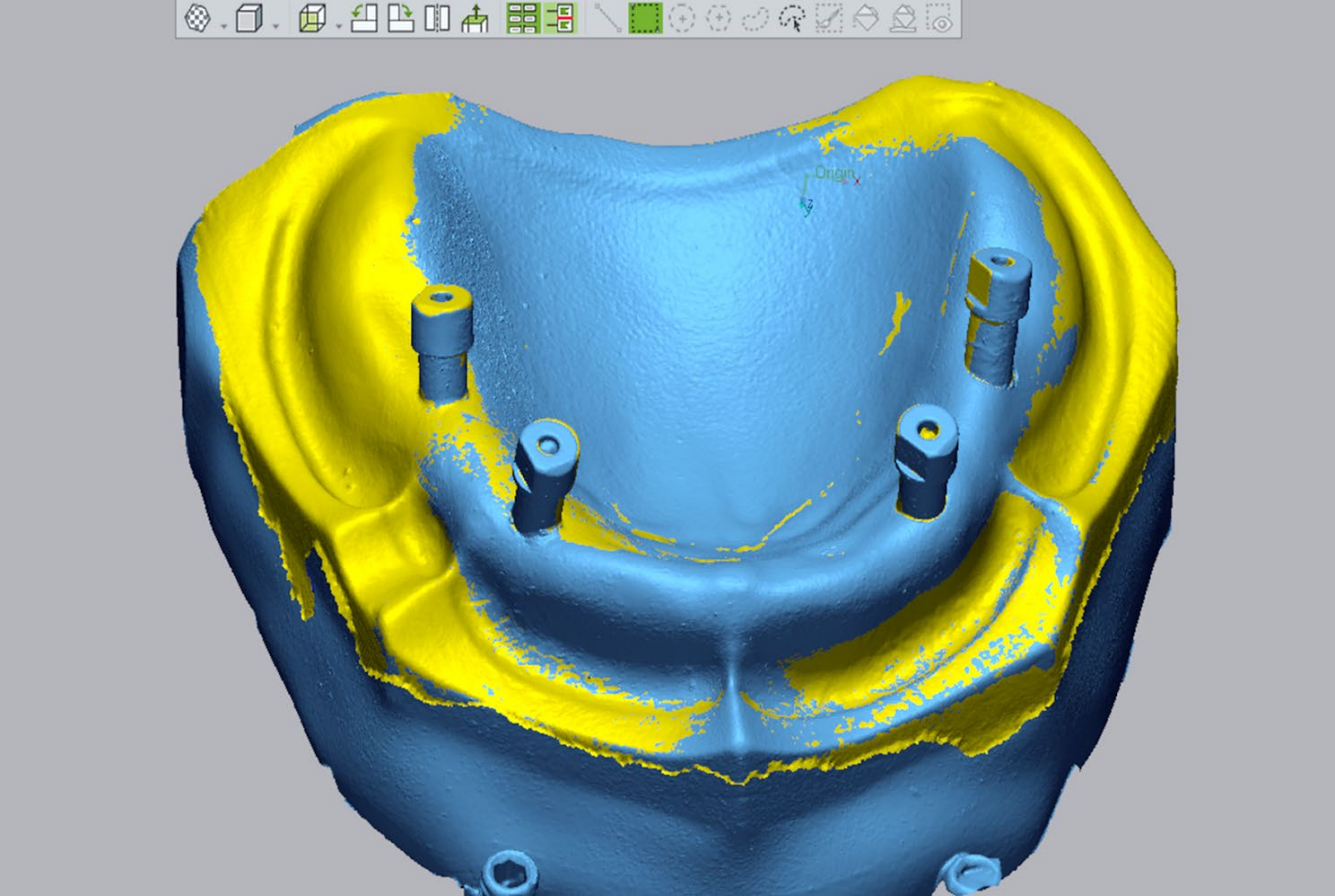
Superimposition between the two digital impressions representing the comparator group and the reference model

**Fig. 5:**
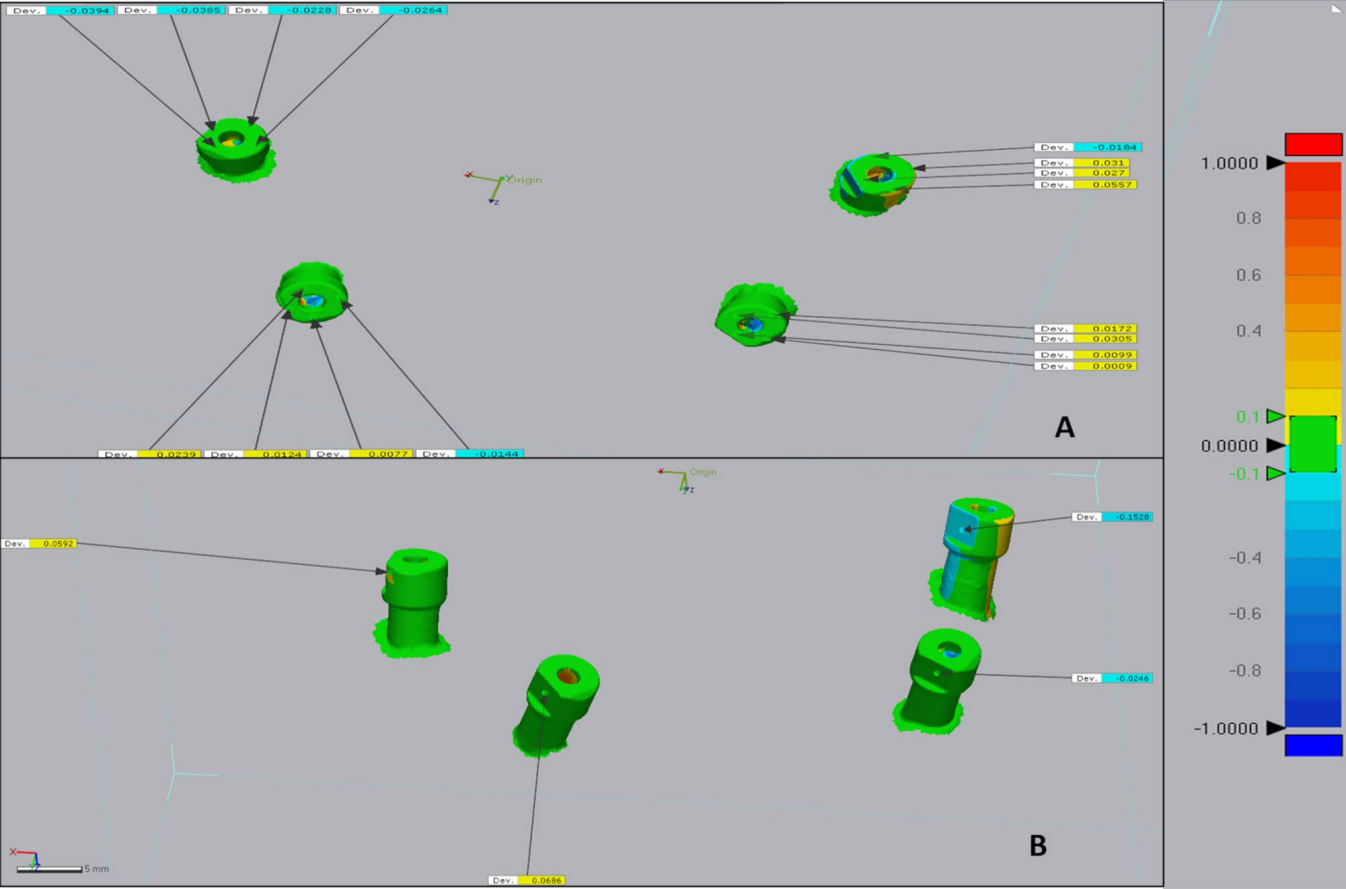
**(A)** Picked up occlusal comparison points on the scan body head **(B)** Picked up mid-axial comparison points on the scan body head

The superimposition step was performed using the initial alignment and the best-fit alignment software tool. Five comparison points were recorded at the buccal, palatal, mesial, and distal aspects of each scan body’s occlusal surface and a mid-axial point at the scan body’s flat surface. (Figures 4 and 5)

**Fig. 6:**
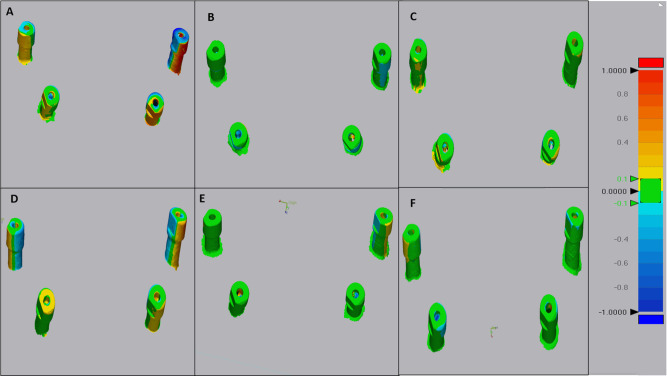
Color map representing trueness and precision in root mean square in the three studied groups **(A)** Trueness of the Lanuca IOS group **(B)** Trueness of the Medit I700 IOS group **(C)** Trueness of the Trios IOS group **(D)** Precision of the Lanuca IOS group **(E)** Precision of the Medit I700 IOS group **(F)** Precision of the Trios IOS group

The accuracy of each intraoral scanner group was represented using a color map of green, yellow, red, and blue colors, where green colors represent zero deviation, blue colors represent negative deviation values, and yellow and red colors represent positive deviation values. These negative and positive values are not true values; they represent direction, as deviation may occur in two opposite directions. The total scan body deviation for each group was represented using the root mean square (RMS.) (Fig. 6).

### Statistical methodology

All the previous data was allocated for study and statistical analysis. The data was explored for normality using the Shapiro-Wilk test and Kolmogorov-Smirnov test, and it was revealed that the significant level (P-value) was shown to be insignificant > 0.05, which indicated that the data originated from a normal distribution (parametric data) in all groups regarding trueness and precision.

Comparisons between the three studied groups were performed using the one-way ANOVA test, followed by Tukey`s post-Hoc test.

## Results

### Trueness evaluation

**Fig. 7:**
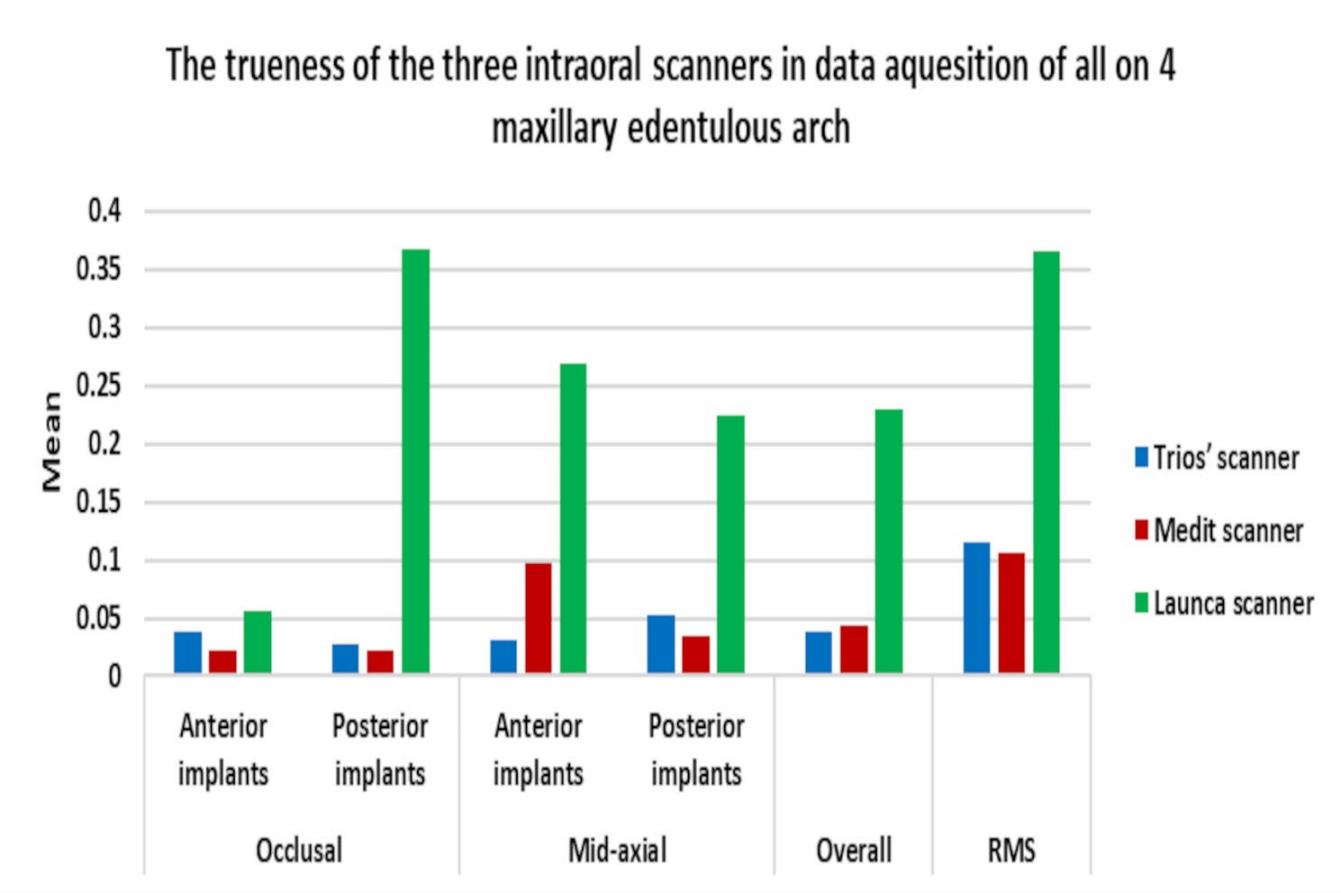
Bar chart representing the trueness of the three digital impression groups in the straight anterior implants and the angled posterior implants

The mean trueness values of each scanner on the occlusal and mid-axial surfaces of anterior and posterior implants were presented in Table 1 and (Fig. 7). Comparison between all groups was performed by using the one-way ANOVA test, which revealed significant differences in all surfaces, followed by Tukey`s post-Hoc test, which revealed that:

The Launca scanner group showed a statistically significant deviation, recording the highest deviation values of 229 μm, while there was no statistically significant difference between the Trios and the Medit groups, recording deviation values of 38 μm and 44 μm, respectively.

### Precision evaluation within each group and comparison between them

**Fig. 8:**
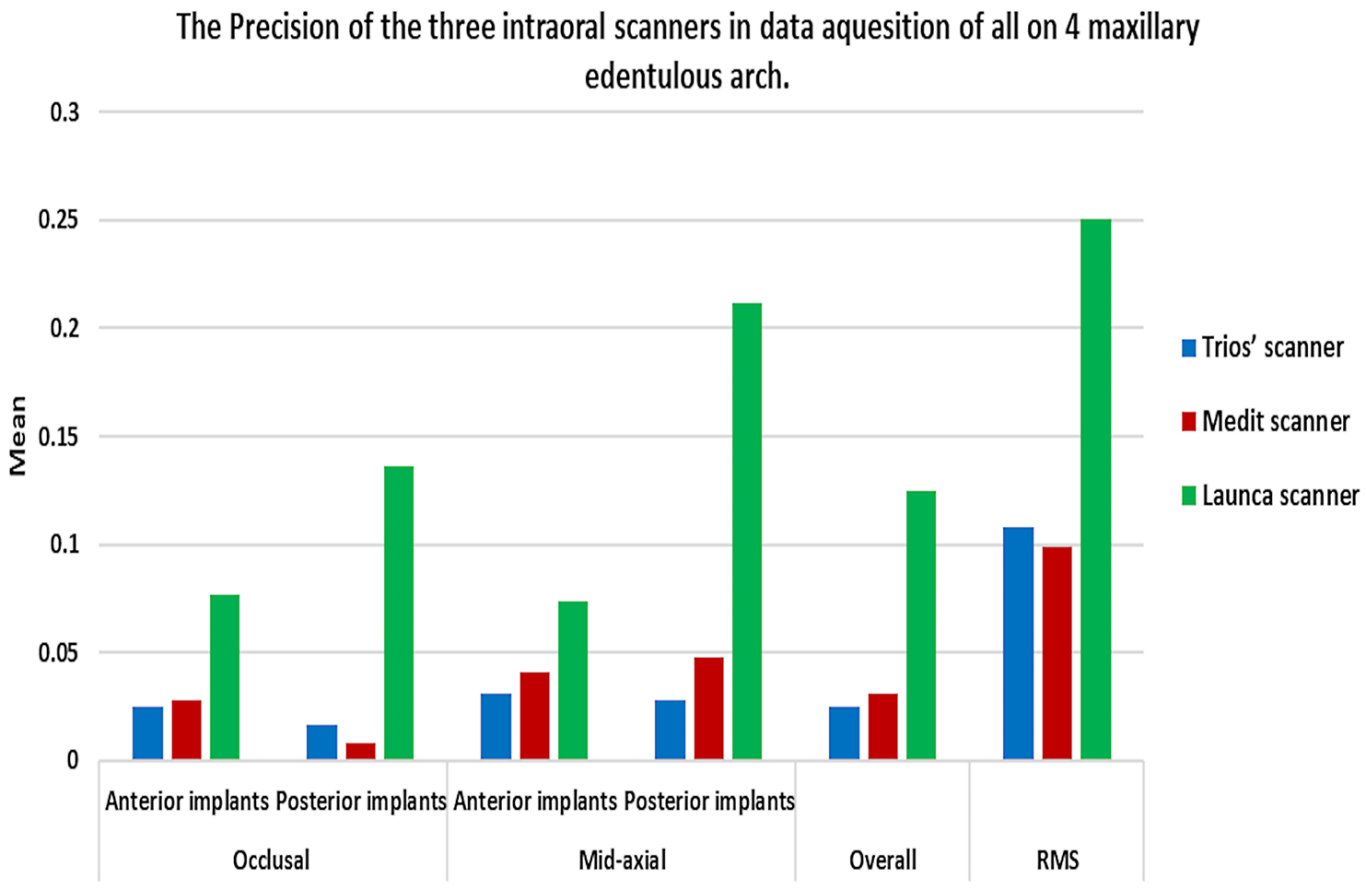
Bar chart representing the precision of the three digital impression groups in the straight anterior implants and the angled posterior implants

The mean precision values of each scanner group were presented in Table 2 and (Fig. 8). The Launca scanner group showed the highest deviation in the three studied groups, recording an overall deviation of 125 μm, and the Trios group showed the lowest deviation values, while there was an insignificant difference between the Trios group and the Medit group regarding the occlusal surface of the anterior implant, the mid-axial surface of the posterior implant, and the RMS.

## Discussion

This study evaluated the accuracy of different intraoral scanners through trueness and precision in the maxillary edentulous arch treated with the All-on-4 concept. The accuracy of digital implant impressions was studied on an in vitro resin model to avoid many complicated surgical procedures of implant placement, such as sinus augmentation or ridge splitting, and their postoperative complications [7, 8, 19, 28]. 

The posterior implants were placed with a 30-degree distal angulation to mimic a real clinical situation in patients, preventing trauma to vital structures and providing better stress distribution, more anchorage, and less cantilever forces at the posterior implants [8, 19, 29]. 

The maxillary edentulous model was 3D printed in photopolymerizing resin because the 3D printing process produces accurate, lightweight, dense, and wear- and damage-resistant models. Additionally, the virtual model data may be stored digitally [30, 31]. 

The Eos x5 desktop scanner was used as a reference for the trueness comparison of the different IOS, as it is a highly precise non-contact optical scanner with an accuracy of 2.8 μm depending on digital stripe projection scanning technology with blue light reference [32, 33]. 

The three intra-oral scanners used in this study were chosen because they use different scanning technologies, originate from different countries, and have different costs. The Trios3 (3 shape) depends on structured light (confocal microscopy) and ultrafast optical scanning [34], while Medit I-700 and Launca depend on triangulation as the technology of acquisition [18]. Many studies stated that different IOSs showed excellent results in terms of trueness and precision in dentulous patients while scanning a completely edentulous patient is still more challenging [19, 34, 35]. 

For standardization, although the three groups can resume scanning once tracking is lost, any cuts or errors during scanning lead to the exclusion of this scan to provide maximum accuracy. Scans were done according to the manufacturer’s instructions for each scanner. All the scans are done without any powdering on the cast.

The Geomagic Control software was utilized in the analysis in this study because, when compared to other inspection software, it showed to have the best level of consistent accuracy [25, 36–38]. 

The model STL file was segmented into 2 segments, as reported by Dohiem et al. [14] as the first segment, including the model, was used for superimposition between the scan data. In contrast, the 2nd segment was the scan bodies, which are the area of comparison, preventing the collection of any irrelevant results from any region other than the scan bodies.[39]

The Medit I-700 and Launca IOS showed more deviation at the mid-axial surface of anterior implants in comparison to posterior implants, recording 97, and 269 μm, respectively. This may be attributed to the short distance between the anterior implant located at the canine area and the posterior implants at the premolar area, which affects the movement of the large scanner head between the implants, leading to a less accurate virtual impression.

The Trios scanner showed no significant difference between the occlusal and mid-axial points of anterior or posterior scan bodies due to its high resolution and accuracy resulting from confocal microscopy technology, recording a non-significant clinical deviation ranging from 25 μm to 31 μm. These results are in agreement with a study conducted by Osman R. and Alharbi N. that concluded after comparing three IOS with different scanning technologies that the Trios scanner has the highest accuracy regarding trueness and precision [18]. 

Although the Medit I700 recorded a 48 μm mid-axial deviation of posterior teeth, it was not clinically significant from the Trios scanner.

According to the results of this in vitro study, there was statistically no significant difference between intra-oral scanners according to the technology of image capture, as Medit I-700 and trios3 depend on triangulation and structured light technology, respectively. Despite Launca dl 202 showing significant deviation and less accuracy through trueness and precision, this confirms the claim that the technology of acquisition has no significant effect on both scanners. On the other hand, a study conducted in 2023 proved that scanning technology affects the accuracy of IOS [18]. 

The Launca scanner showed highly significant deviation points of 397 μm in the occlusal surface of posterior teeth and 269,224 μm in the mid-axial of anterior and posterior implants, respectively. These high deviation values of the Launca scanner may be attributed to the old design of the scanner head and body.

This study tested the accuracy of different intraoral scanners on only an in-vitro edentulous maxillary model, which is considered a limitation as many clinical factors that affect the accuracy of the scanner were not evaluated, such as the presence of saliva, tongue mobility, and cheeks and lip. Also, the scanning of all on 4 implants on a mandibular arch was not evaluated as mandibular scanning is challenging. Also, the software manipulation feature of each scanner wasn’t tested and evaluated in this study. Another limitation of this study is the comparison of the accuracy of the intra-oral scanner with other data acquisition methods such as photogrammetry.

## Conclusions

within the limitations of this in-vitro study evaluating the accuracy of three intra-oral scanners on scanning four implants in the maxillary edentulous arch:

- The Trios scanner was the most accurate, followed by the Medit I-700, and the Launca scanner showed the highest deviation of the three scanners.
- The scanner technology of data acquisition doesn’t affect the scanning accuracy as the scanner head design does.
- The Trios scanner has the ability to scan the posterior angulated implants as accurately as the anterior straight ones.

**Table 1:** Mean and standard deviation of trueness in all scanners and comparison between them

Trueness		Trios’ scanner		Medit scanner		Launca scanner		*P* value
		M	SD	M	SD	M	SD	
Occlusal	Anterior implants	0.038 ^**a**^	0.026	0.022 ^**a**^	0.015	0.057 ^**b**^	0.062	0.0284*
	Posterior implants	0.028 ^**a**^	0.015	0.023 ^**a**^	0.021	0.367 ^**b**^	0.212	0.0001*
Mid-axial	Anterior implants	0.032 ^**a**^	0.028	0.097 ^**a**^	0.037	0.269 ^**b**^	0.086	0.0001*
	Posterior implants	0.053 ^**a**^	0.050	0.035 ^**a**^	0.030	0.224 ^**b**^	0.106	0.0001*
Overall		0.038 ^**a**^	0.015	0.044 ^**a**^	0.011	0.229 ^**b**^	0.078	0.0001*
RMS		0.116 ^**a**^	0.021	0.106 ^**a**^	0.016	0.366 ^**b**^	0.120	0.0001*

Min: minimum Max: maximum

M: mean SD: standard deviation

*Significant difference as *P* < 0.05

Means with the same superscript letters were insignificantly different as *P* > 0.05

Means with different superscript letters were significantly different as *P* < 0.05

**Table 2:** Mean and standard deviation of precision in all scanners and comparison between them

Precision		Trios’ scanner		Medit scanner		Launca scanner		*P* value
		M	SD	M	SD	M	SD	
Occlusal	Anterior implants	0.025 ^**a**^	0.020	0.028 ^**a**^	0.015	0.077 ^**b**^	0.037	0.004*
	Posterior implants	0.017 ^**a**^	0.021	0.008 ^**a**^	0.008	0.136 ^**a**^	0.161	0.058
Mid-axial	Anterior implants	0.031 ^**a**^	0.016	0.041 ^**ab**^	0.022	0.0735 ^**b**^	0.027	0.01*
	Posterior implants	0.028 ^**a**^	0.032	0.048 ^**a**^	0.022	0.212 ^**b**^	0.193	0.026*
Overall		0.025 ^**a**^	0.009	0.031 ^**a**^	0.014	0.125 ^**b**^	0.084	0.005*
RMS		0.108 ^**a**^	0.018	0.099 ^**a**^	0.034	0.251 ^**b**^	0.109	0.002*

Min: minimum Max: maximum

M: mean SD: standard deviation

*Significant difference as *P* < 0.05

Means with the same superscript letters were insignificantly different as *P* > 0.05

Means with different superscript letters were significantly different as *P* < 0.05

## Data Availability

No datasets were generated or analysed during the current study.
